# Increase in the incidence of *Candida parapsilosis* and *Candida tropicalis* bloodstream infections during the coronavirus disease 2019 (COVID-19) pandemic

**DOI:** 10.1017/ash.2022.354

**Published:** 2023-01-10

**Authors:** Thanus Pienthong, Suttichai Visuttichaikit, Piyaporn Apisarnthanarak, Kittiya Jantarathaneewat, David J. Weber, Anucha Apisarnthanarak

**Affiliations:** 1 Division of Infectious Diseases, Faculty of Medicine, Thammasat University, Pathum Thani, Thailand; 2 Division of Diagnostic Radiology, Department of Radiology, Faculty of Medicine Siriraj Hospital, Mahidol University, Bangkok, Thailand; 3 Center of Excellence in Pharmacy Practice and Management Research, Faculty of Pharmacy, Thammasat University, Pathum Thani, Thailand; 4 Research Group in Infectious Diseases Epidemiology and Prevention, Faculty of Medicine, Thammasat University, Pathum Thani, Thailand; 5 Division of Infectious Diseases, University of North Carolina, Chapel Hill, North Carolina, United States

*To the Editor—*Nosocomial candidemia is associated with substantial mortality, longer hospital stays, and higher healthcare costs. The mortality among patients with candidemia remains high and is associated with increasing in incidence of non–*Candida albicans Candida* spp.^1,2^ Previous studies have suggested that non–*C. albicans* candidemia has increased during the coronavirus disease 2019 (COVID-19) pandemic, probably due to the increasing use of central venous catheters, suboptimal catheter care, and concurrent corticosteroid use.^3–5^ *Candida parapsilosis* bloodstream infection (BSI) has been associated with the overuse of central venous catheters and receipt of parenteral nutrition, and risk factors for *Candida tropicalis* are poorly defined.^6,7^ Overall, the *C. parapsilosis* and *C. tropicalis* BSI incidence rates at Thammasat University Hospital increased from 0.42% to 2.24% and from 1.68% to 7.46% between 2019 and 2021, respectively, whereas the rate of *C. albicans* BSI remained stable at 6.70%–7.83%. We performed a case–case–control study to identify risk factors for and outcomes of *C. parapsilosis* and *C. tropicalis* BSIs at Thammasat University Hospital (Pathum Thani, Thailand), a tertiary-care center.

For the period from January 1, 2019, through December 31, 2021, we evaluated the risk factors and outcomes of *C. parapsilosis* and *C. tropicalis* BSIs compared with *C. albicans* BSI using the case 1–case 2–control method. Study participants were identified from the microbiology laboratory database, which includes all positive blood cultures for all *Candida* spp. Case 1 was defined as patients with *C. parapsilosis* BSI. Case 2 was defined as patients with *C. tropicalis* BSI. Controls were patients with *C. albicans* BSI. A BSI was defined as isolation of the *Candida* species of interest from at least 1 peripheral venous sample or central venous sampling. Blood samples were processed using an automated BACTEC-NR system (Becton Dickinson, Franklin Lakes, NJ). Thereafter, the *Candida* spp were identified using CHROMagar *Candida* (CHROMagar, Paris, France) for *C. albicans* and *C. tropicalis* and a VITEK-2 identification card (bioMèrieux, Marcy-l’Étoile, France) for *C. parapsilosis.*

Data collected included demographics, source of candidemia based on medical records, risk factors for *Candida* BSI, APACHE II score, antifungal therapy, adequate source control, infection prevention bundles for insertion and maintenance of central lines based on hospital IC database, duration of catheterization, and crude mortality. Observation of insertion and maintenance bundles based on the hospital’s infection control policy were performed by infection prevention nurses using checklists according to the Asia Pacific Society of Infection Control recommendations.^8^

All analyses were performed using SPSS version 26 software (IBM, Armonk, NY). We used χ^2^ tests to compare categorical variables. Independent *t* tests were used for continuous data. All *P* values were 2-tailed, and *P* < .05 was considered statistically significant. A multivariate analysis was conducted to evaluate factors and outcomes associated with *C. parapsilosis* and *C. tropicalis* BSI compared to *C. albicans*. We calculated adjusted odd ratios (aORs) and 95% confidence intervals (CIs). The outcomes included crude in-hospital mortality, clinical cure, and microbiological cure.

During the study period, 69 patients were identified with a *Candida* BSI: 9 (13%) with *C. parasilopsis*, 20 (29%) with *C. tropicalis*, and 40 (58%) with *C. albicans*. The rate of *C. parapsilosis* BSI was 2.23 BSIs per 10,000 blood cultures. The rate of *C. tropicalis* BSI was 7.46 BSIs per 10,000 blood cultures, and the rate of *C. albicans* BSI was 7.82 BSIs per 10,000 blood cultures. The median patient age was 66 years (range, 1–95 years). Also, 21 (30.4%) of the 69 patients in the study cohort were oncologic patients, and all received home parenteral nutrition during the COVID-19 pandemic. Risk factors and outcomes for *C. parapsilosis* and *C. tropicalis* BSI are summarized in Table 1. Patients with a *C. parasilopsis* BSI had a higher proportion of indwelling catheters than patients with a *C. albicans* BSI (Table 1). The duration of catheter use was longer in cases of *C. tropicalis* BSI than in cases of *C. albicans* BSI (22.5 ± 13.0 days vs 18.6 ± 8.0 days; *P* = .02). To investigate insertion and maintenance process of care, observations for CVCs or PICC lines revealed only 25% full compliance with insertion and maintenance bundles. Among 69 infection prevention observations, 59 (85.5%) identified failure to comply with maximal sterile barrier precautions, 20 (28.9%) showed improper disinfection of catheter hubs, 15 (21.7%) revealed improper dressing or dressing leaks, and 25 (36.2%) showed damp or loosened dressings that were retained. Notably, the use of a 3-way stopcock connected to the IV catheter hub was noted in 17 (24.6%) of these 69 observations.

**Table 1.** Characteristics and Outcomes of the Patients With *C. parapsilosis* and *C. tropicalis* BSI Compared With Patients With *C. albicans* Bloodstream Infection

Variables	*C. parapsilosis* (n = 9)	*C. albicans* (n = 40)	*P* Value	*C. tropicalis* (n = 20)	*C. albicans* (n = 40)	*P* Value
Age, median y (IQR)	45 (1–90)	68 (2–95)	.06	64 (23–91)	68 (2–95)	.39
Sex, male, no. (%)	4 (44.4)	20 (50)	.77	13 (65)	20 (50)	.28
**Comorbidities, no. (%)**						
DM	3 (33.3)	12 (30)	.85	5 (25)	12 (30)	.69
Gastrointestinal disease	2 (22.2)	2 (5)	.09	6 (30)	2 (5)	.01
Malignancy	2 (22.2)	14 (35)	.47	5 (25)	14 (35)	.44
Immunocompromised	1 (11.1)	9 (22.5)	.45	5 (25)	9 (22.5)	.83
Others^a^	0 (0)	18 (45)	.08	10 (50)	18 (45)	.84
**Source of candidemia, no. (%)**						
CLABSI	7 (77.8)	27 (67.5)	.55	14 (70)	27 (67.5)	.85
Intraabdominal	1 (11.1)	5 (12.5)	.91	5 (25)	5 (12.5)	.23
Urinary tract	0 (0)	8 (20)	.15	1 (5)	8 (20)	.13
Unknown	1 (11.1)	5 (12.5)	.91	1 (5)	5 (12.5)	.10
APACHE II score, median (IQR)	11.0 (2–20)	16.5 (2–26)	.18	14 (4–32)	16.5 (2–26)	.30
**Risk factor, no. (%)**						
Indwelling CVCs/PICCs	7 (77.8)	28 (70)	.64	14 (70)	28 (70)	1.00
Parenteral nutrition	5 (55.6)	11 (27.5)	.11	5 (25)	11 (27.5)	.84
Urinary catheter	4 (44.4)	36 (90)	.002	18 (90)	36 (90)	1.00
Mechanical ventilator	3 (33.3)	31 (77.5)	.01	14 (70)	31 (77.5)	.53
Duration of central venous catheter insertion, median d (IQR)	19.5 (12–27)	18.6 (10.6–26.6)	.08	22.5 (9.5–35.5)	18.6 (10.6–26.6)	.02
Total antifungal duration, median d (IQR)	17 (3–53)	9.5 (0–79)	.05	5 (0–25)	9.5 (0–79)	.64
Adequate antifungal therapy, no. (%)	8 (88.9)	28 (70)	.25	16 (80)	28 (70)	.41
Source control, no. (%)	8 (88.9)	24 (60)	.10	9 (45)	24 (60)	.28
Proper, no. (%)^b^	2 (28.6)	5 (17.9)	.53	3 (21.4)	5 (17.9)	.78
Crude in-hospital mortality, no. (%)	2 (22.2)	27 (67.5)	.01	16 (80)	27 (67.5)	.32
Clinical cure, no. (%)	7 (77.8)	13 (32.5)	.01	4 (20)	13 (32.5)	.32
Microbiological cure, no. (%)	9 (100)	27 (67.5)	.04	12 (60)	27 (67.5)	.57

Note. IQR, interquartile range; BSI, bloodstream infection; CLABSI, central-line–associated bloodstream infection; DM, diabetes mellitus; CVC; central venous catheter, PICC; peripherally inserted central catheter; aOR, adjusted odds ratio; CI, confidence interval. By multivariable analysis: (1) a risk factor for *C. parasilopsis* BSI was the receiving of parenteral nutrition (aOR, 4.77; 95% CI, 0.78–29.26); (2) risk factors for *C. tropicalis* BSI included the patient with gastrointestinal disease (aOR, 7.13; 95% CI 1.19–45.64) and the admission in the medical intensive care unit (aOR, 4.01; 95% CI, 0.82–19.72); and (3) a protective factor for mortality for *C. parasilopsis* BSI (aOR, 0.02; 95% CI, 0.01–0.27) and *C. tropicalis* BSI (aOR, 0.03; 95% CI, 0.01–0.29) was the appropriate source control.

a Cardiovascular disease, pulmonary disease, and neurological disease.

b Proper intravascular catheter care includes promptly remove any intravascular catheter that is no longer required and proper dressing and proper dressing change.

Our study has yielded several important findings. First, the incidence of non–*C*. *albicans* candidemia has increased. This finding is consistent with a previous study,^3^ and this increase probably occurred due to the overuse of antibiotics, concurrent corticosteroids, and/or immunomodulatory agents.^9^ Second, we observed prolonged catheter duration and a suboptimal level of compliance with IPC policies, particularly for maintenance bundles. Our data support the important role of maintenance catheter care for long-term catheter use during the COVID-19 pandemic. Although the recommendation for a maintenance bundle for CLABSI has been reinforced by the APSIC, the translation of these recommendation into actual practice has remained suboptimal in Asia.^10^

This study had several limitations. First, we use a retrospective design, and the relatively small sample size limited our ability to identify other risk factors. Second, the nature of a single-center study limits the generalizability of our results to other settings. Despite these limitations, our findings reinforce the important role of the maintenance bundles to help reduced CLABSIs due to non–*C. albicans* spp during the COVID-19 pandemic.
